# Supplementary material for: Fine tuning a logical model of cancer cells to predict drug synergies: combining manual curation and automated parameterization
Source: Front Syst Biol. 2023 Nov 20;3:1252961. doi: 10.3389/fsysb.2023.1252961 (PMC12341965; doi:10.3389/fsysb.2023.1252961)
Supplement: Supplementary file 1 [file Table1.DOCX]

# Supplementary Figures

| 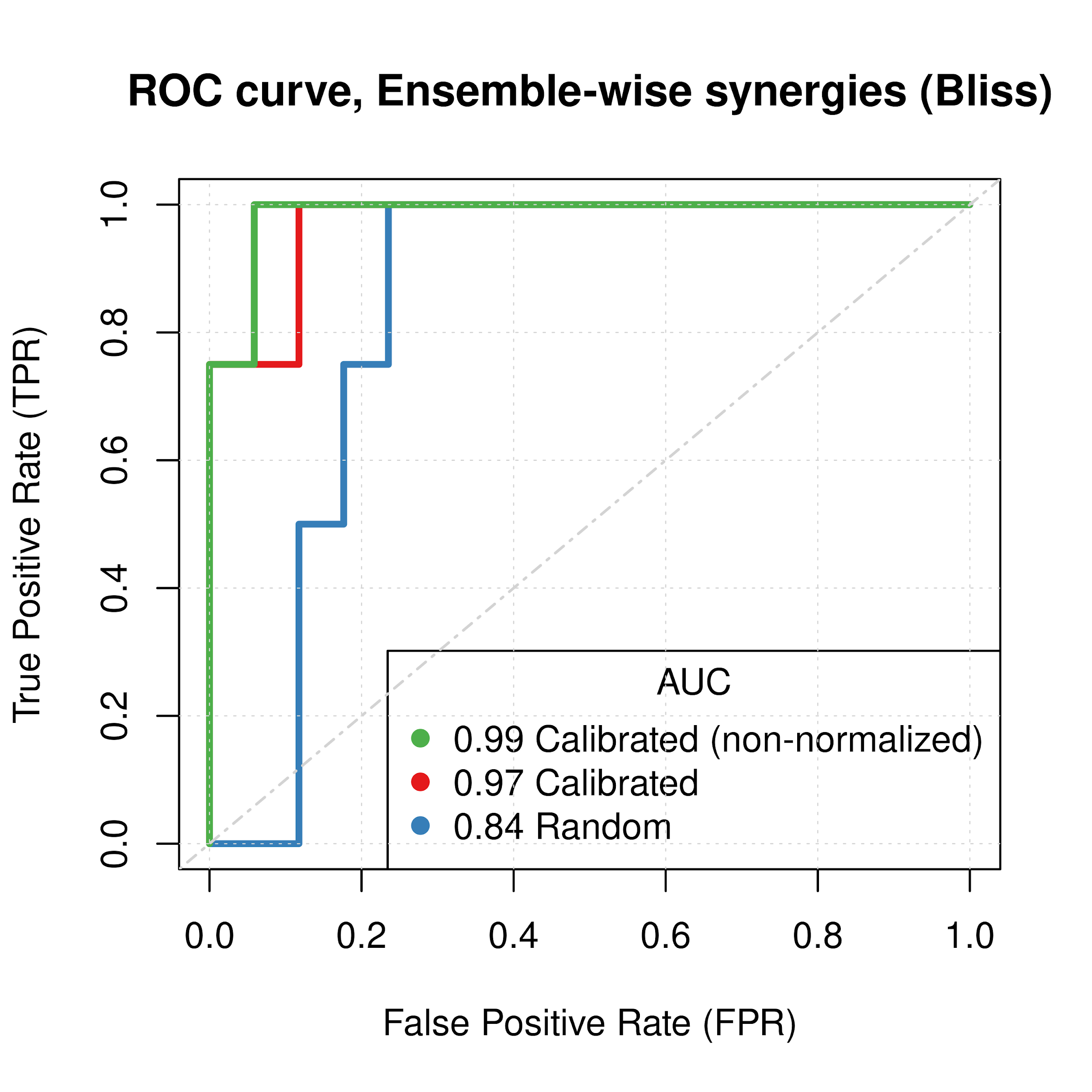 | 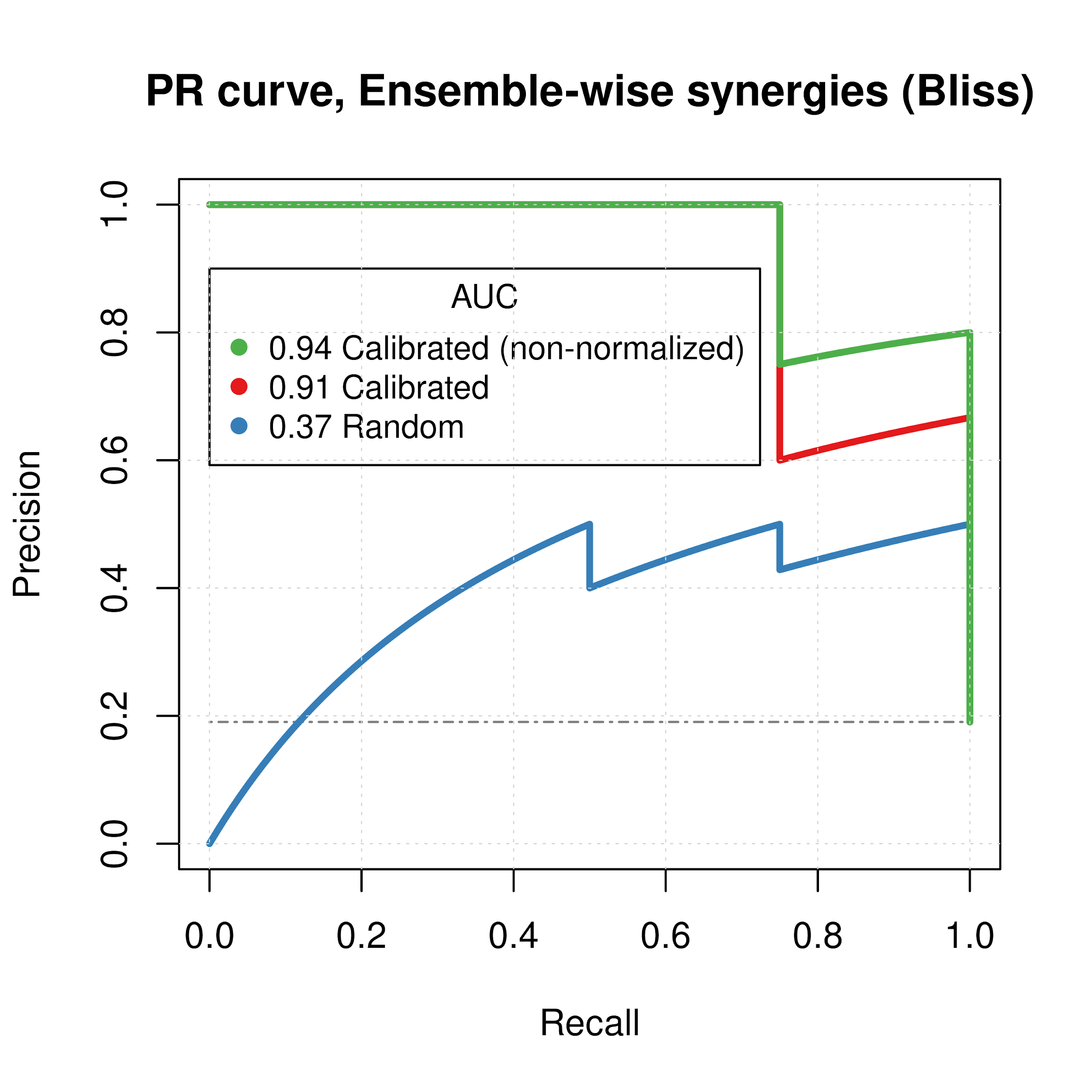 |
| --- | --- |

*Figure S1: Predictive performance for random (proliferative) models, calibrated models (non-normalized) and calibrated normalized to random models (CASCADE 1.0 topology).*

| 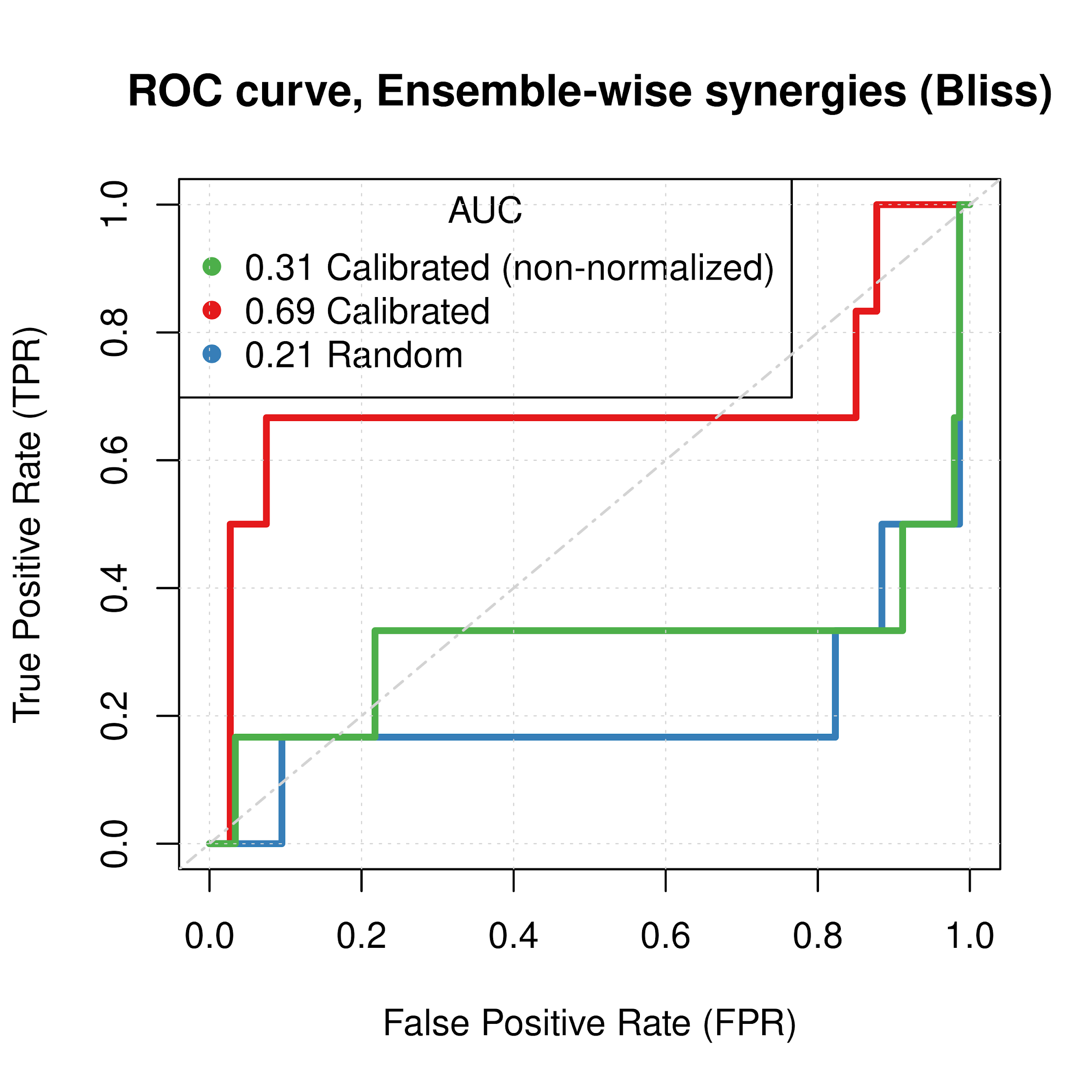 | 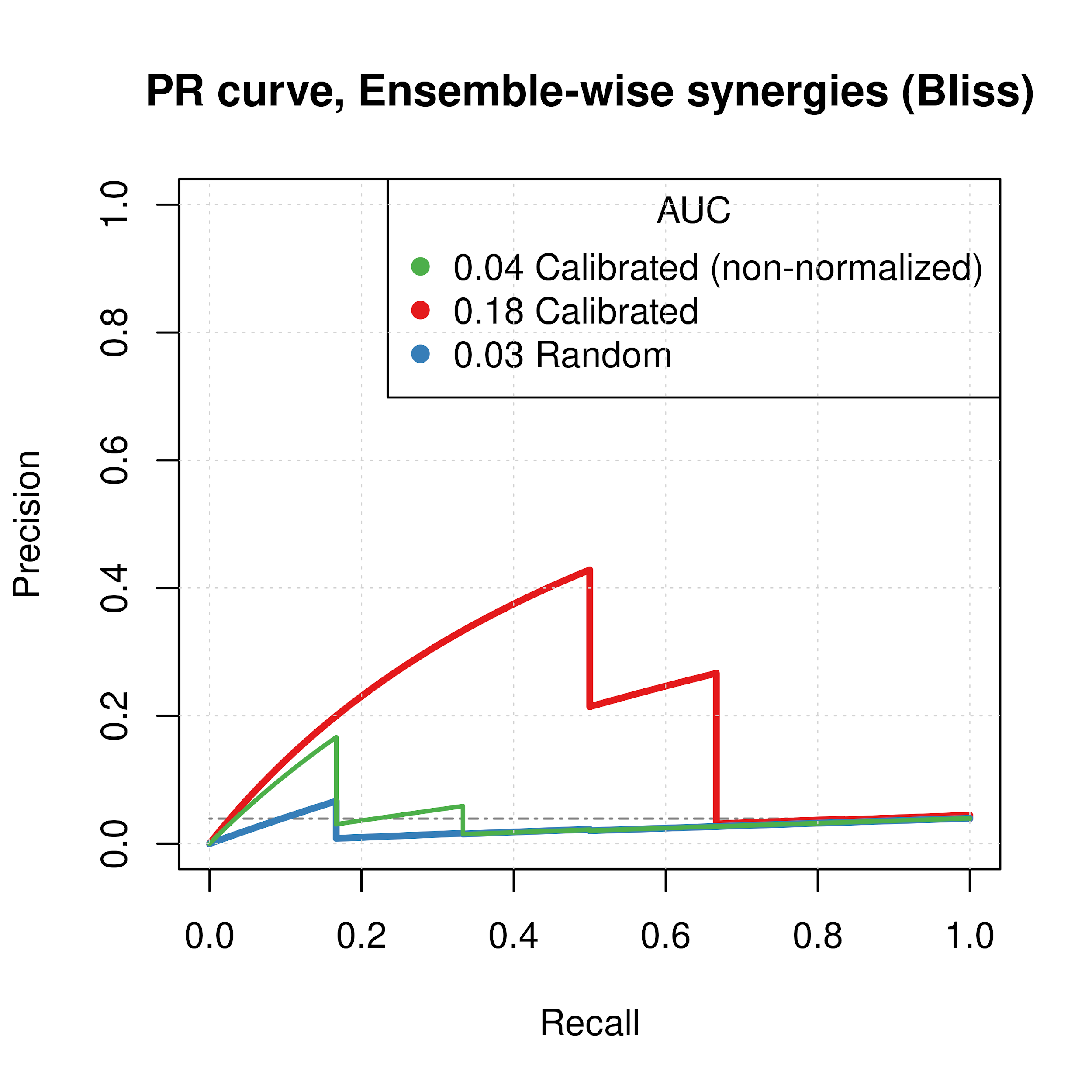 |
| --- | --- |

*Figure S2: Predictive performance for random (proliferative) models, calibrated models (non-normalized) and calibrated normalized to random models (CASCADE 2.0 topology).*


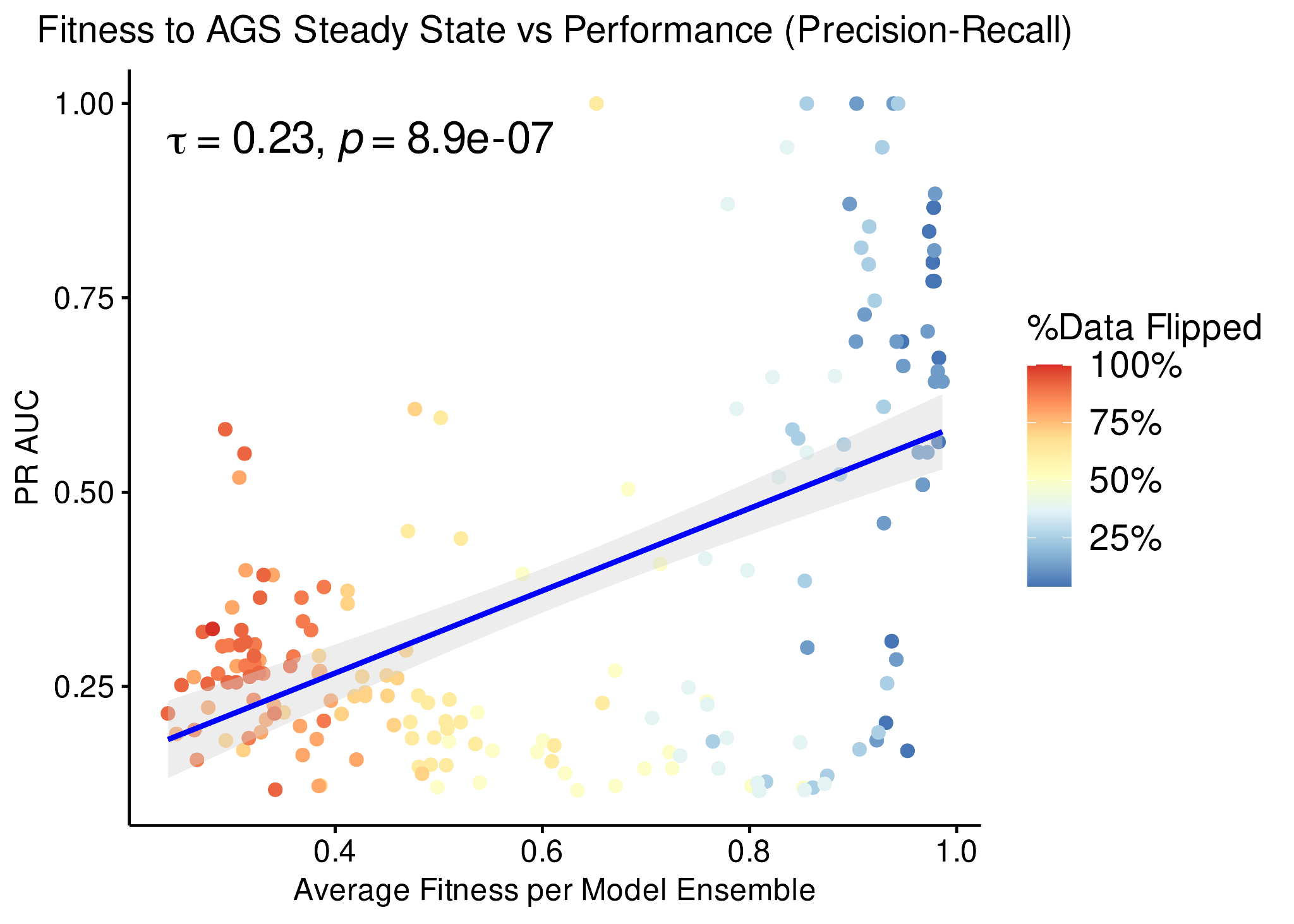


*Figure S3: PR AUC performance dependence on fitness (CASCADE 1.0 topology).*

| 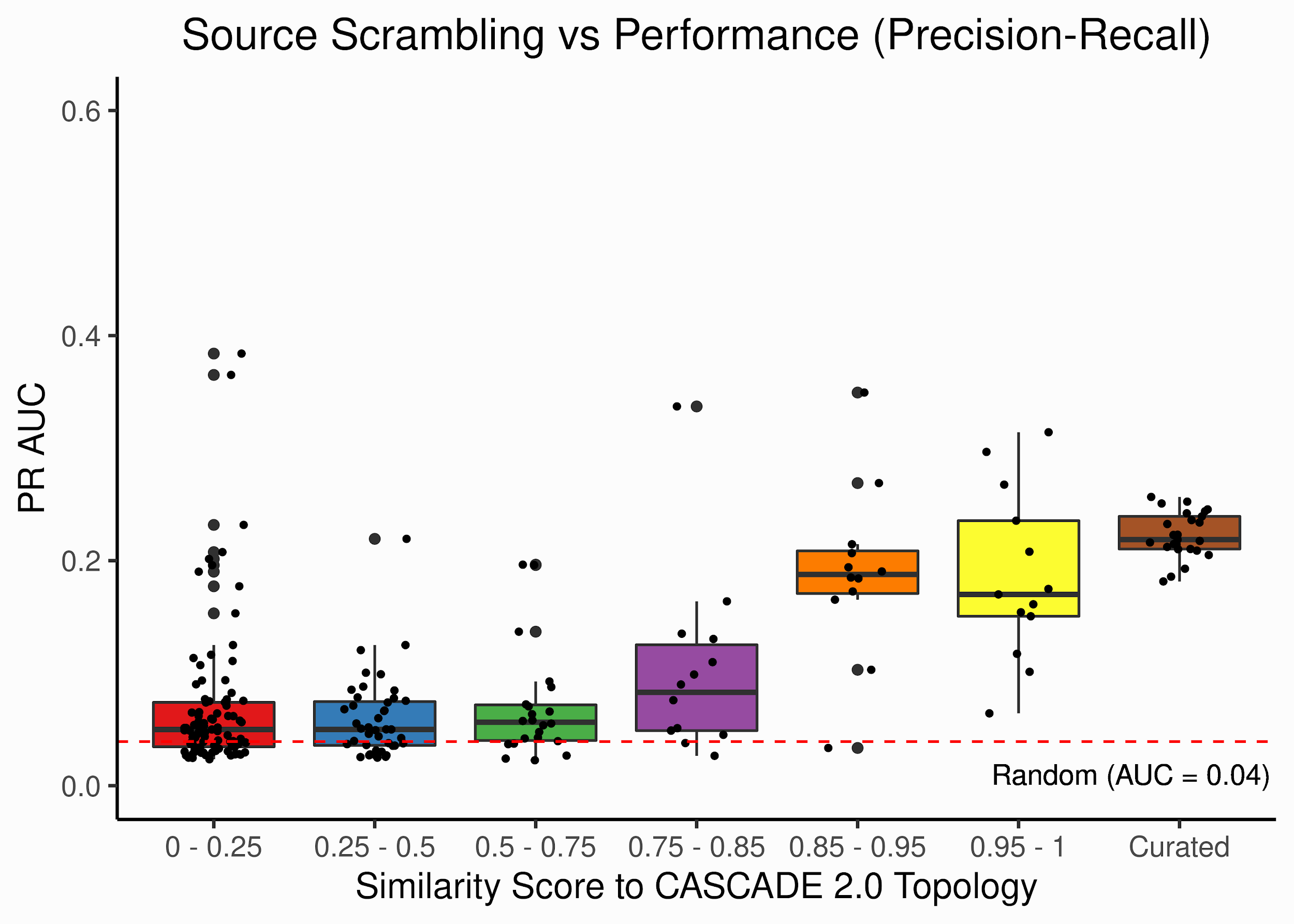 | 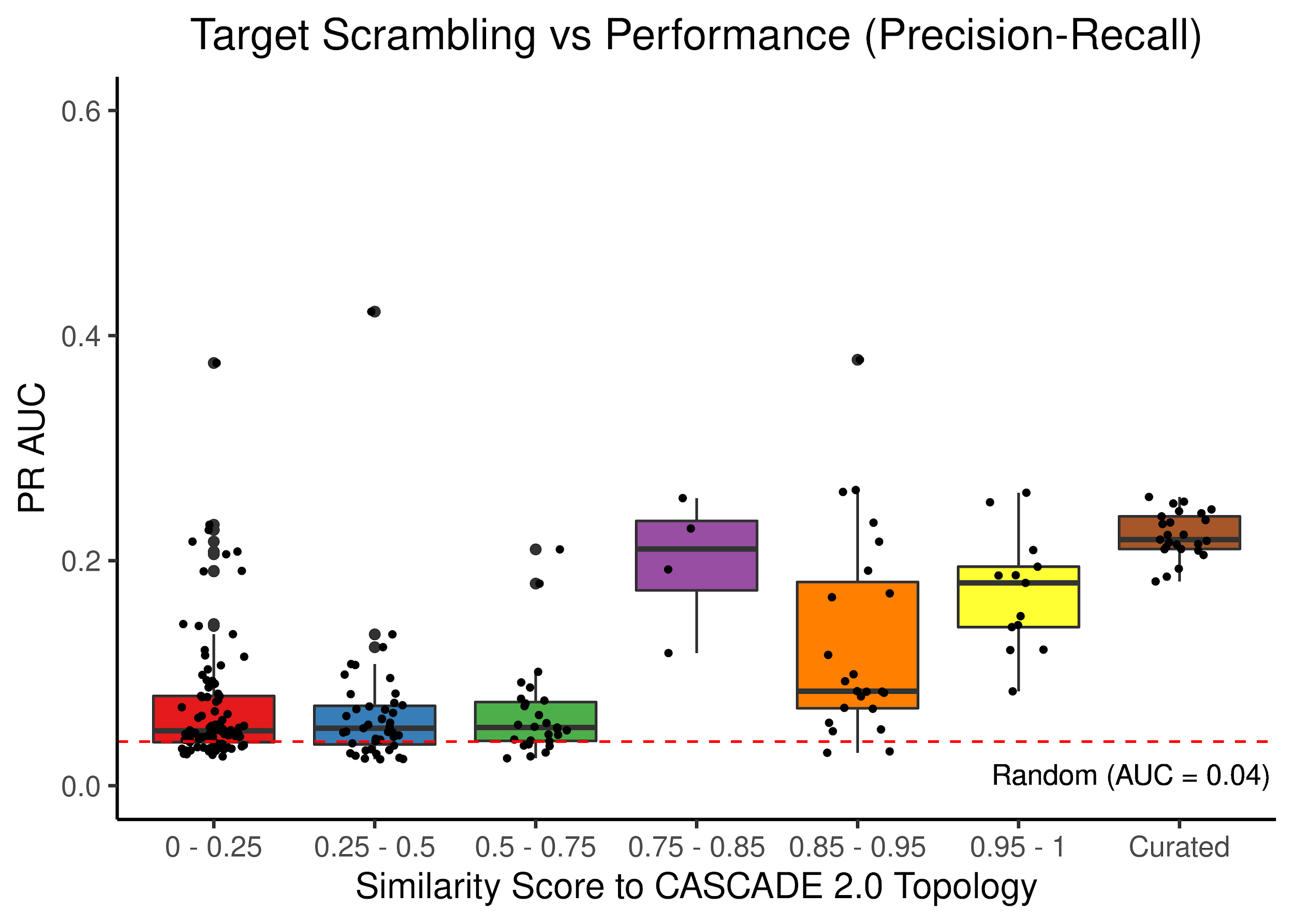 |
| --- | --- |
| 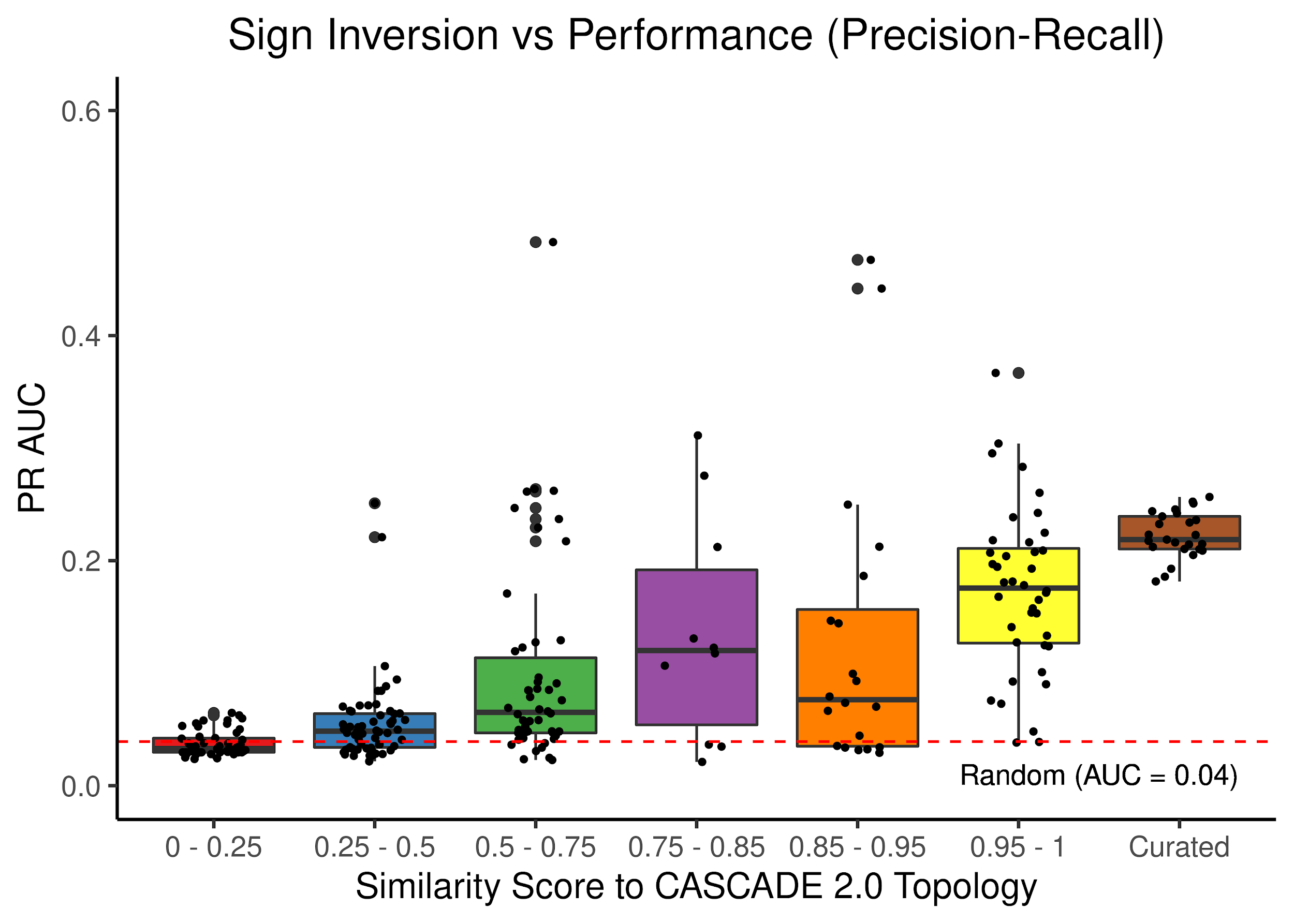 | 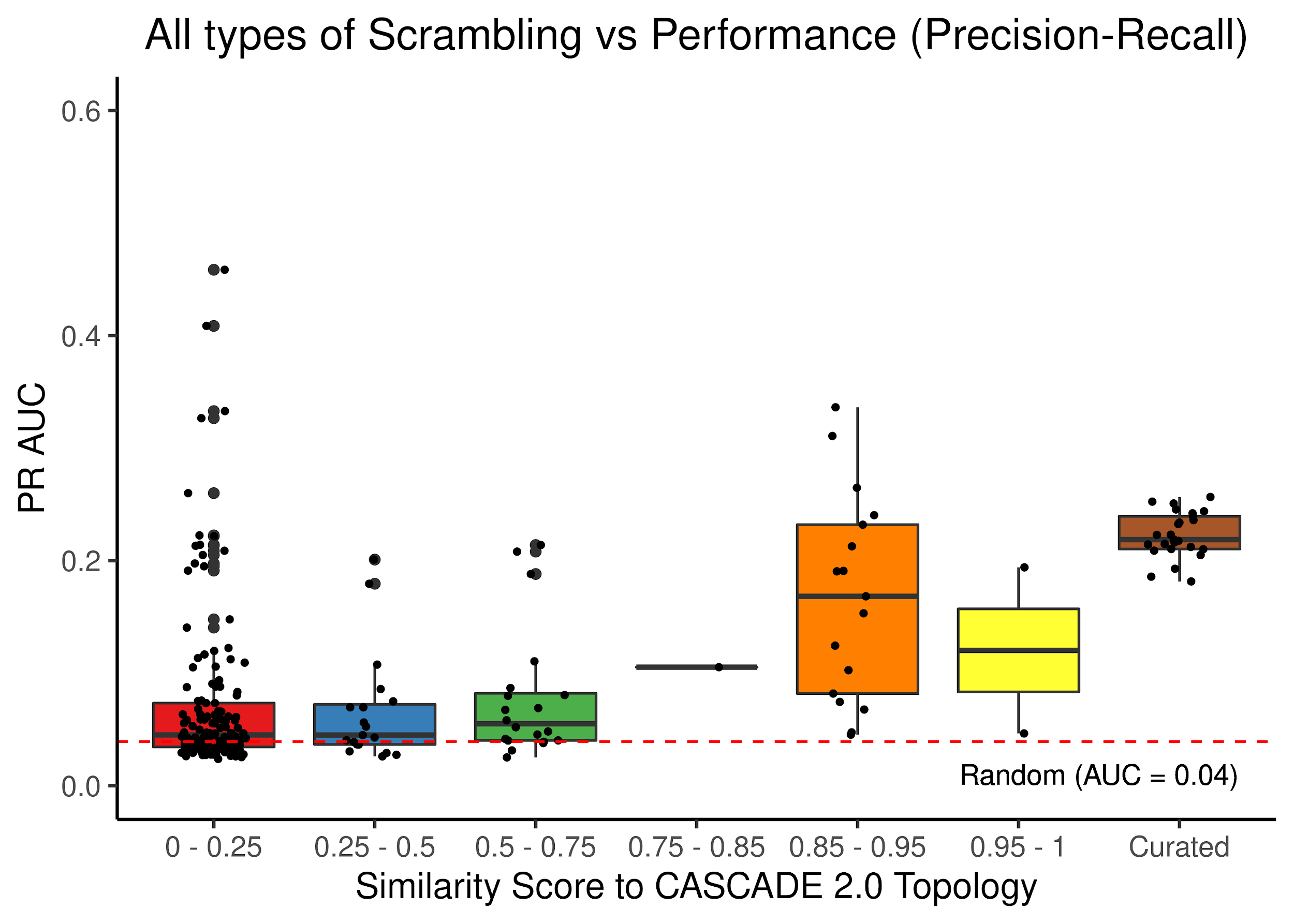 |

*Figure S4: Effects of variations introduced in the CASCADE 2.0 prior knowledge graph (PR AUC performance metric).*

| 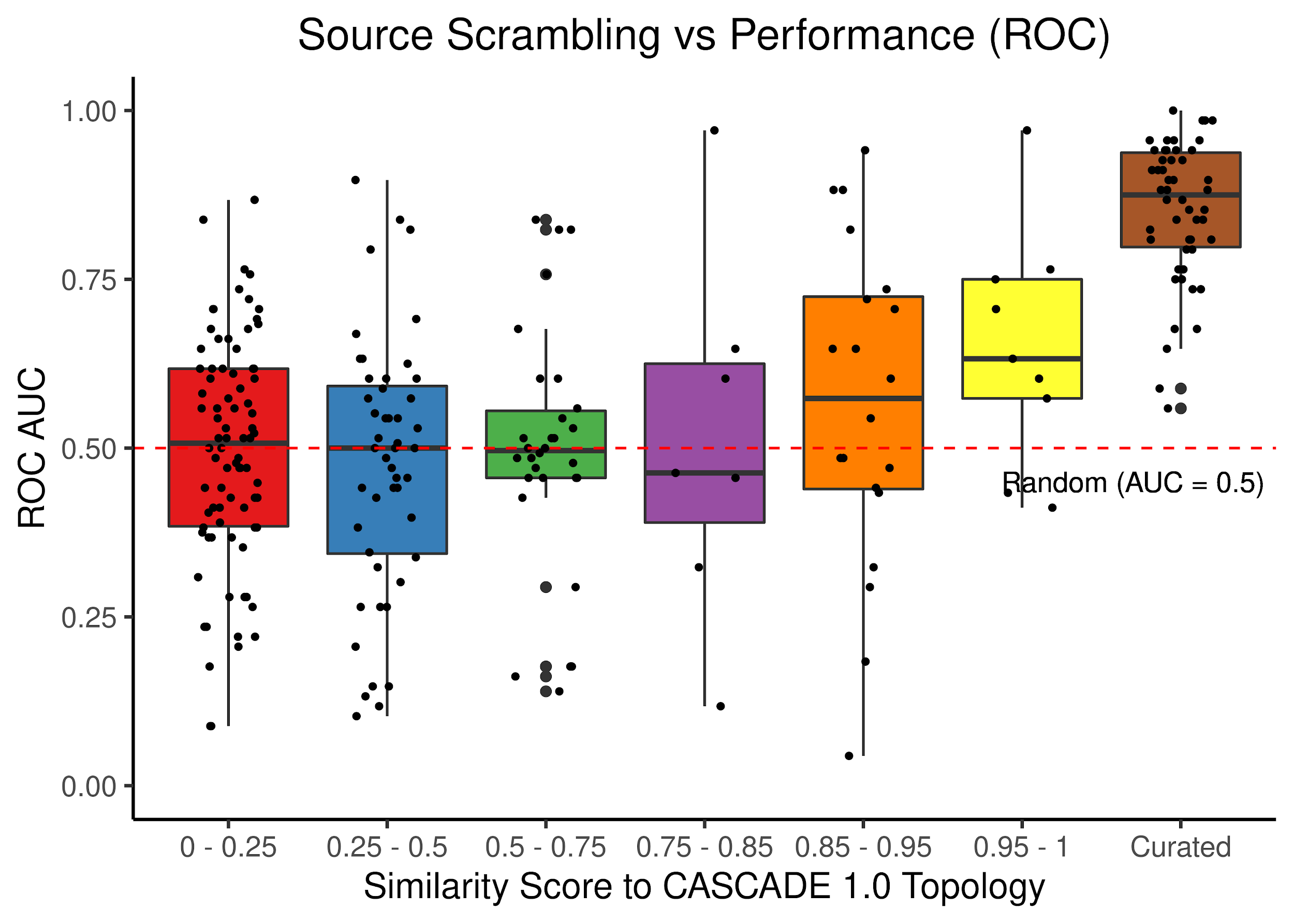 | 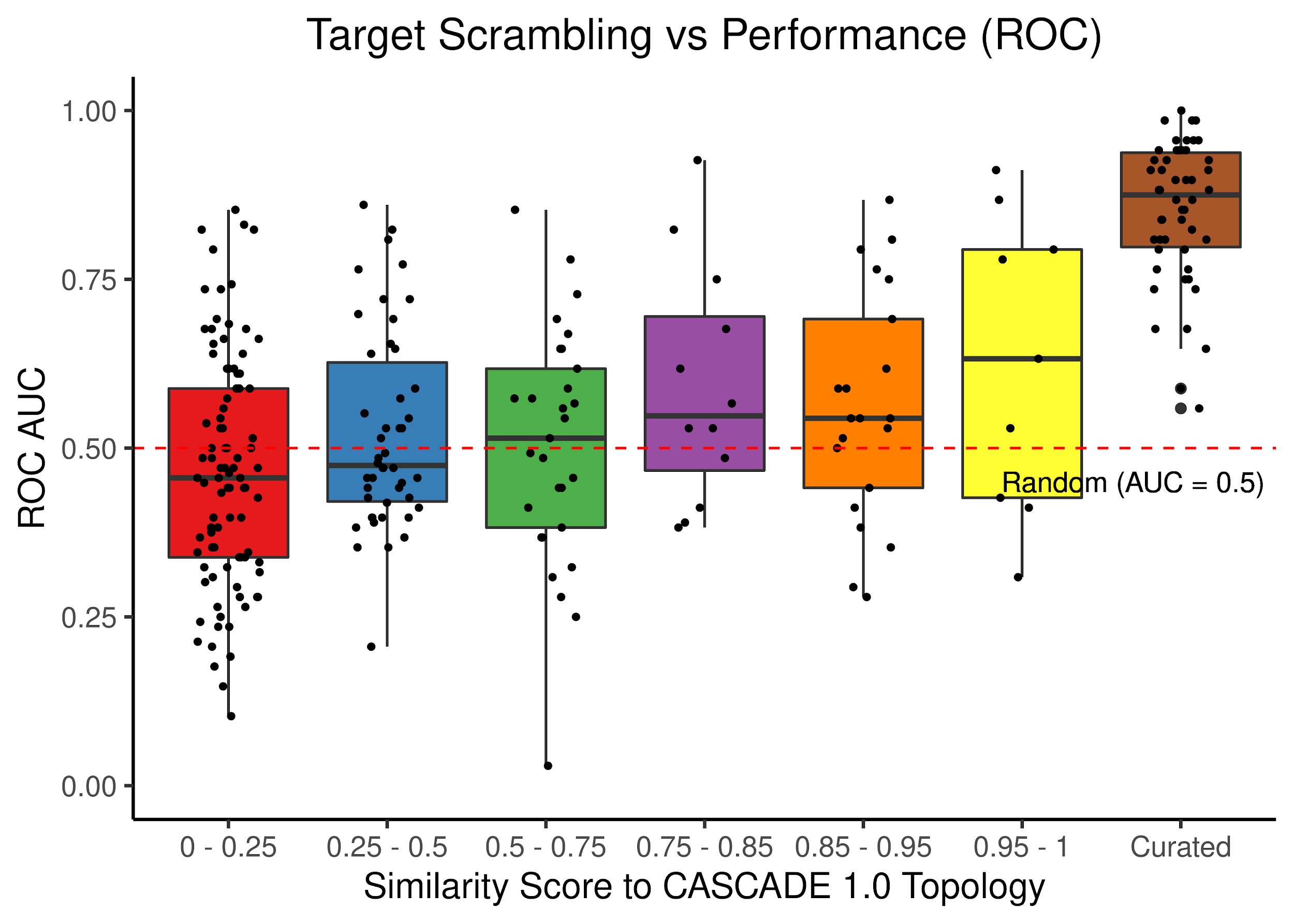 |
| --- | --- |
| 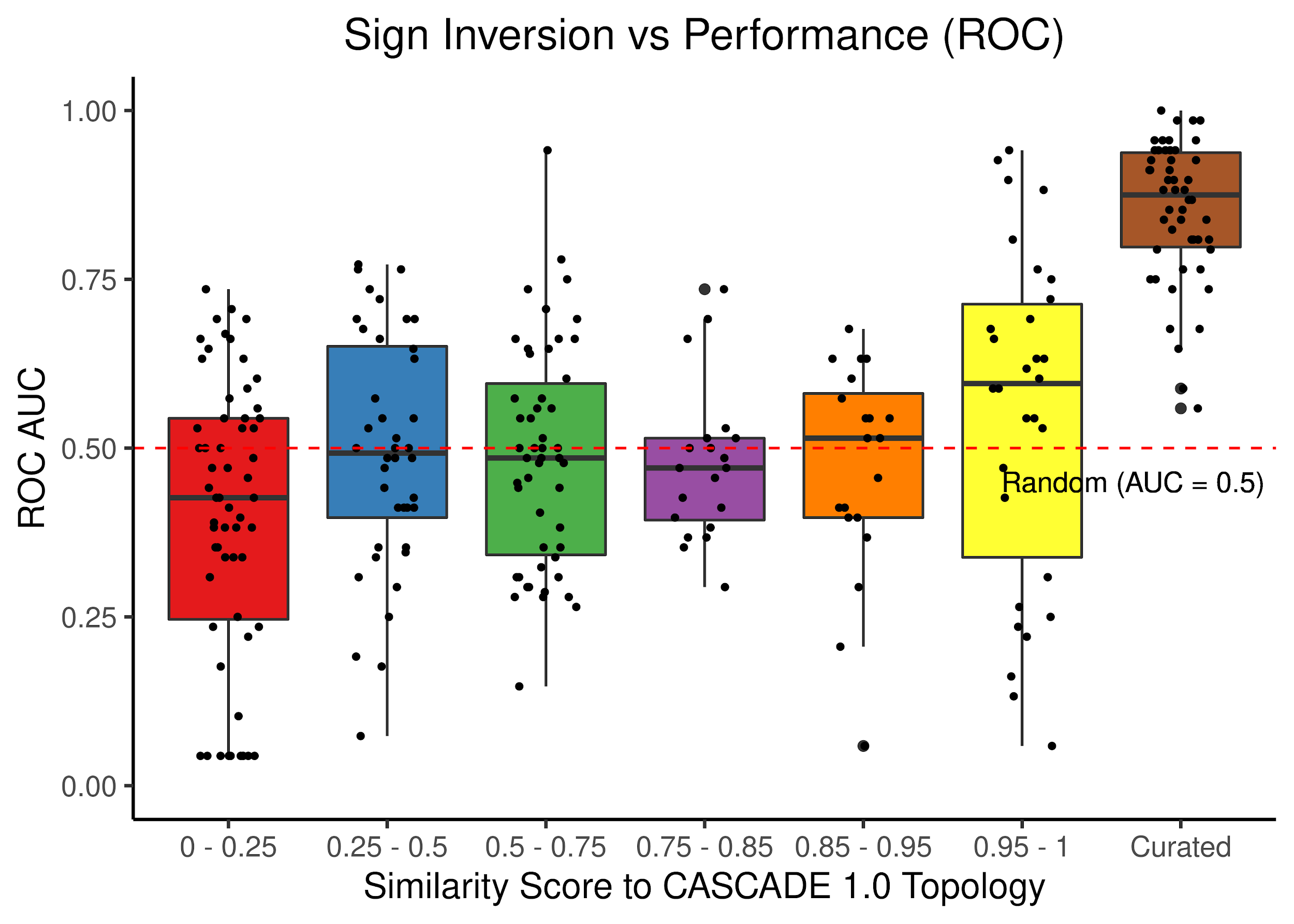 | 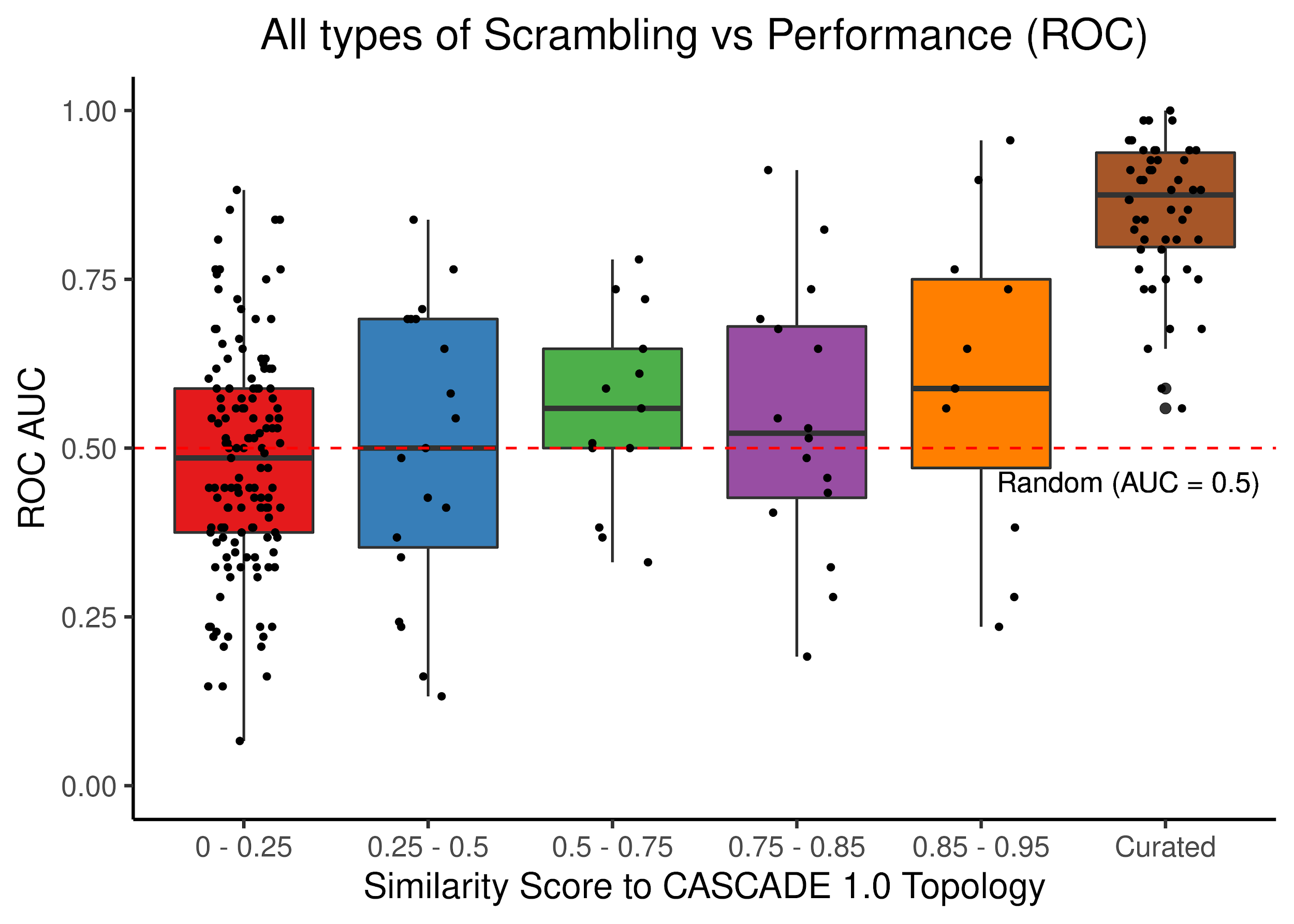 |

*Figure S5: Effects of variations introduced in the CASCADE 1.0 prior knowledge graph (ROC AUC performance metric).*

| 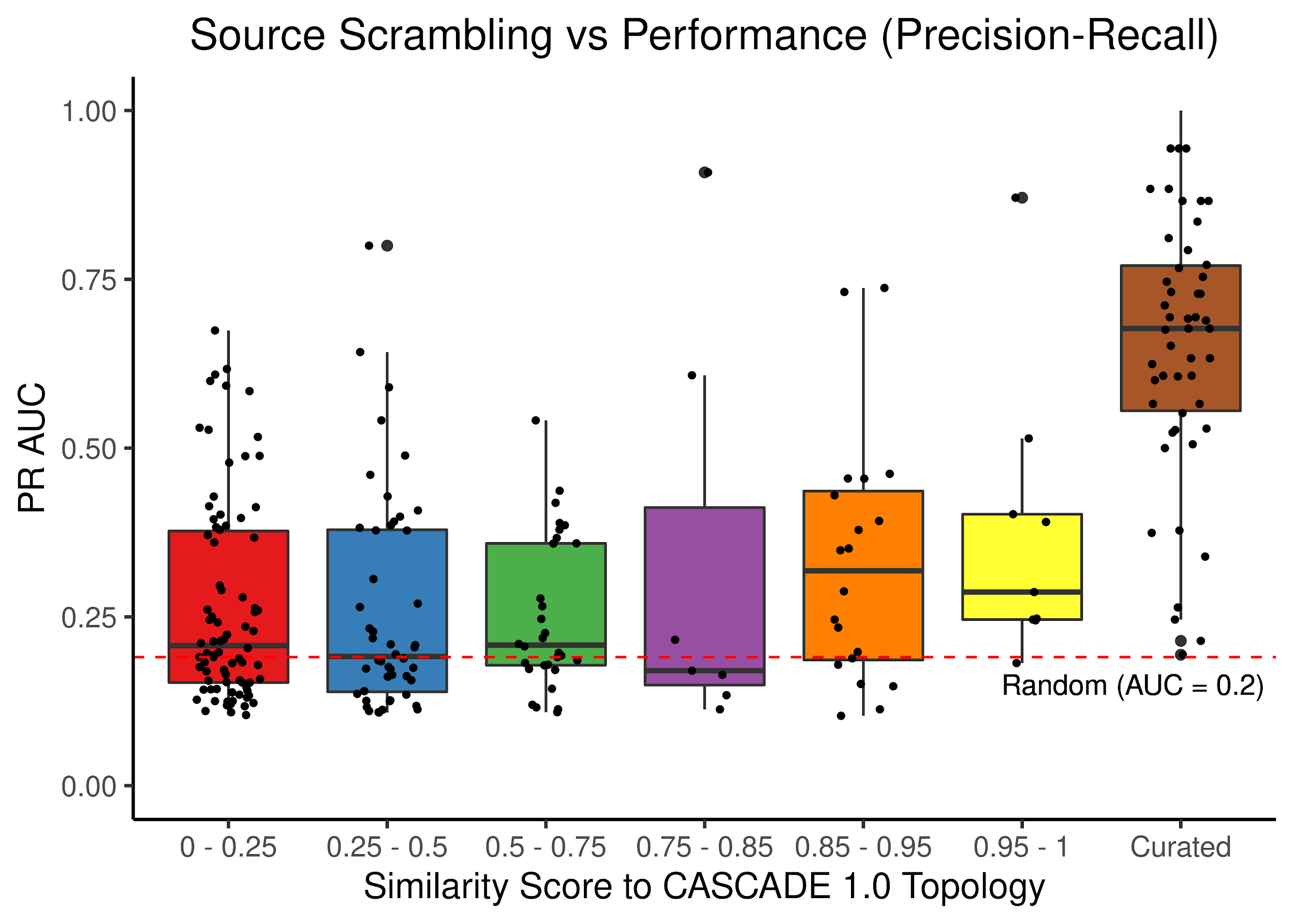 | 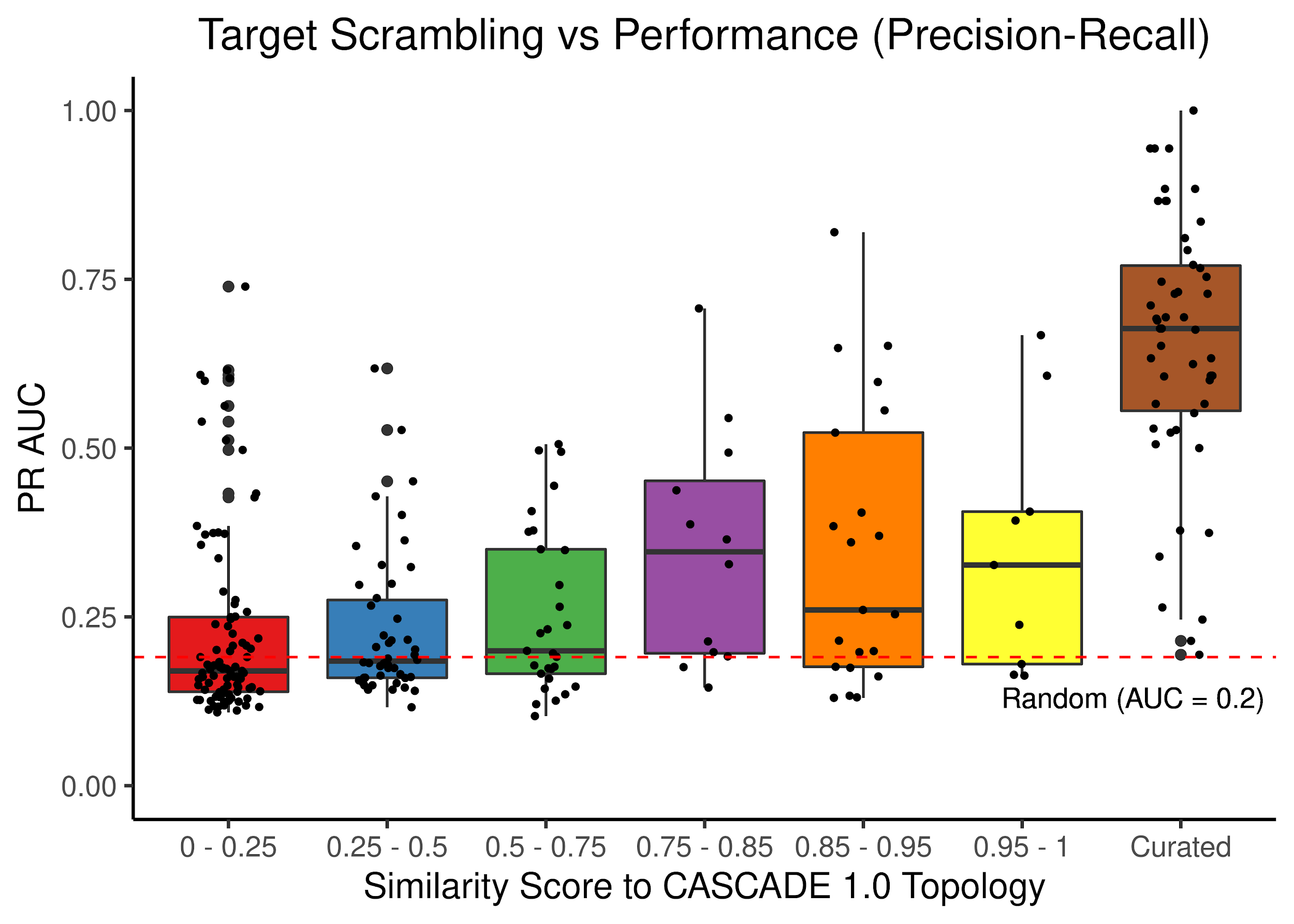 |
| --- | --- |
| 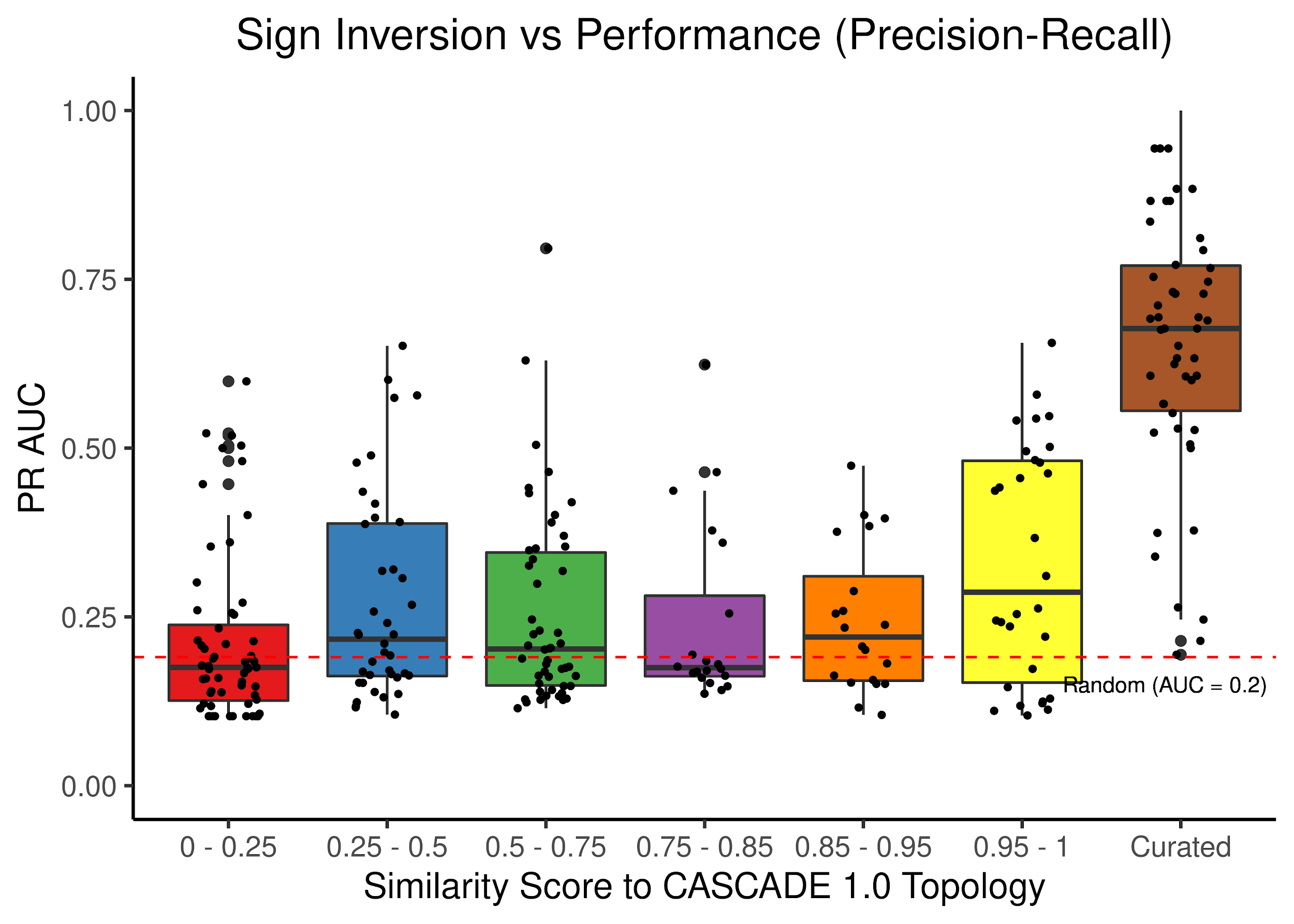 | 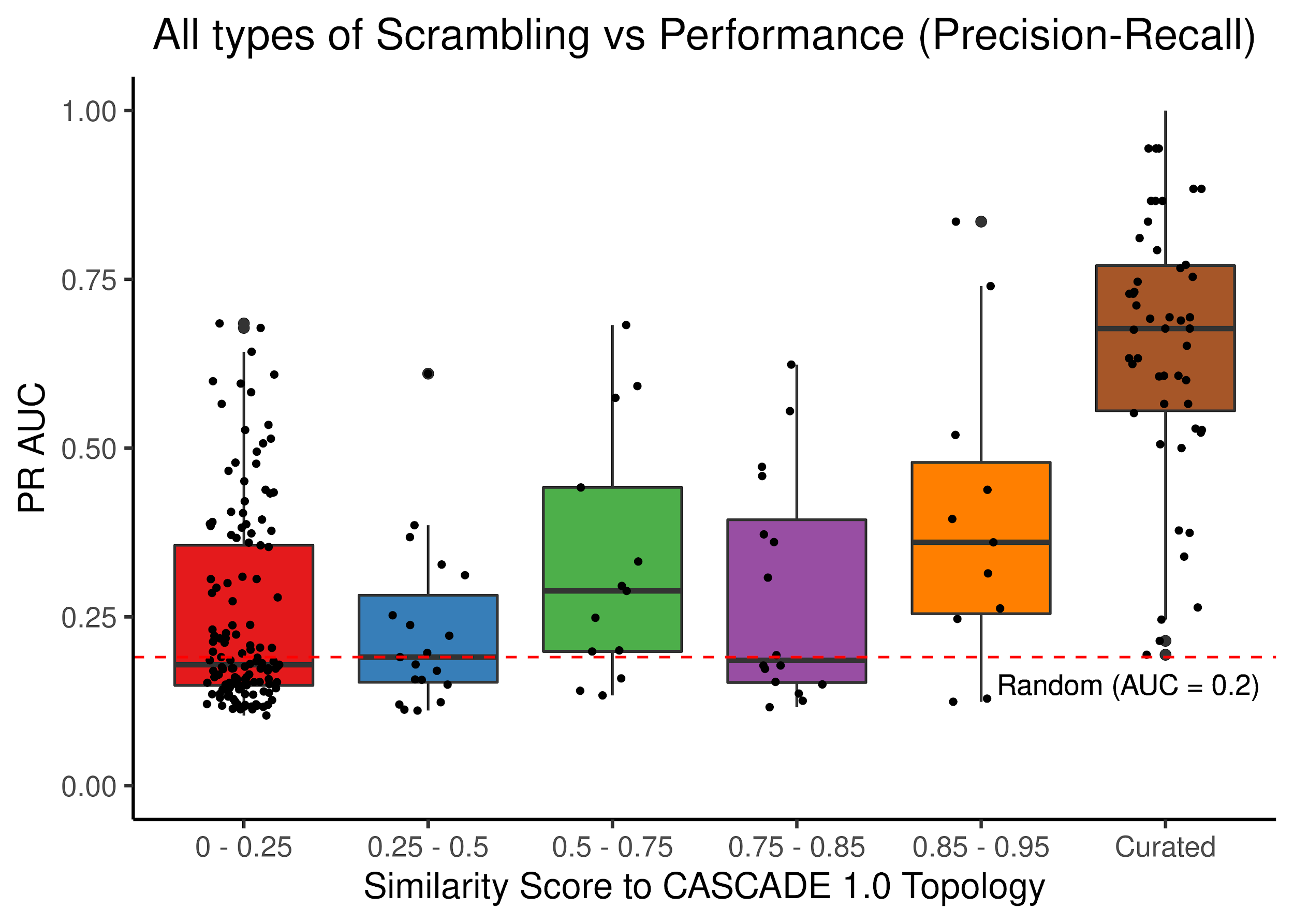 |

*Figure S6: Effects of variations introduced in the CASCADE 1.0 prior knowledge graph (PR AUC performance metric).*

*
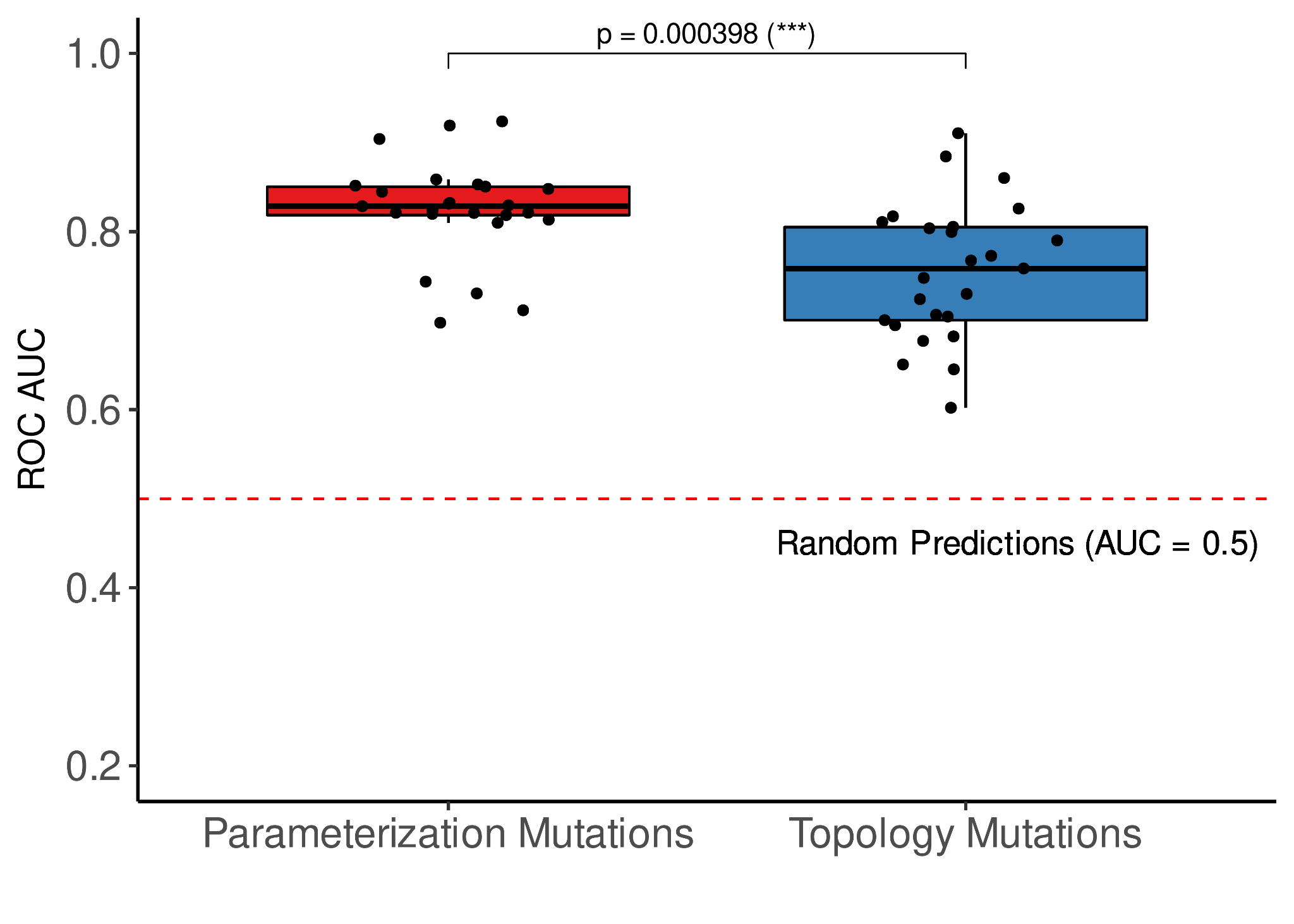
*

*Figure S7: Model performance measured using ROC AUC after parameter and topology modifications.*
